# Quantitative optical mapping of two-dimensional materials

**DOI:** 10.1038/s41598-018-23922-1

**Published:** 2018-04-23

**Authors:** Bjarke S. Jessen, Patrick R. Whelan, David M. A. Mackenzie, Birong Luo, Joachim D. Thomsen, Lene Gammelgaard, Timothy J. Booth, Peter Bøggild

**Affiliations:** 1 Center for Nanostructured Graphene (CNG), Technical University of Denmark, DK-2800, Kgs, Lyngby, Denmark; 2 Department of Micro- and Nanotechnology (DTU Nanotech), Technical University of Denmark, DK-2800, Kgs, Lyngby, Denmark

## Abstract

The pace of two-dimensional materials (2DM) research has been greatly accelerated by the ability to identify exfoliated thicknesses down to a monolayer from their optical contrast. Since this process requires time-consuming and error-prone manual assignment to avoid false-positives from image features with similar contrast, efforts towards fast and reliable automated assignments schemes is essential. We show that by modelling the expected 2DM contrast in digitally captured images, we can automatically identify candidate regions of 2DM. More importantly, we show a computationally-light machine vision strategy for eliminating false-positives from this set of 2DM candidates through the combined use of binary thresholding, opening and closing filters, and shape-analysis from edge detection. Calculation of data pyramids for arbitrarily high-resolution optical coverage maps of two-dimensional materials produced in this way allows the real-time presentation and processing of this image data in a zoomable interface, enabling large datasets to be explored and analysed with ease. The result is that a standard optical microscope with CCD camera can be used as an analysis tool able to accurately determine the coverage, residue/contamination concentration, and layer number for a wide range of presented 2DMs.

## Introduction

The discovery that atomically thin two-dimensional (2D) materials could be visualised optically has arguably been the single most important enabler for the explosive emergence of 2D materials research^1^. On suitable substrates, such as thin SiO_2_ on Si, the optical contrast of, e.g. single-layer graphene can be as high as 14%, which enables the identification of individual layers using standard white-light microscopy. Optical microscopy - while still used to hunt down individual monolayers of mechanically exfoliated 2D crystals for fundamental research and prototyping^2^ - is now also a crucial tool for process development inspection and quality control within large-area graphene growth and transfer processes^3–5^.

Despite remarkable progress in the quality of both growth^6–11^ and transfer^3–5,12,13^ of 2D materials, the atomic thinness and high surface area-to-volume ratio of these materials make them exceptionally susceptible to non-uniformities such as contamination, atomic and mechanical defects, and secondary layer growth, which in turn is reflected in key performance metrics^14^ such as electrical conductivity^15^, charge carrier mobility^16^, and barrier properties^17^. Understanding the origin of such variations and monitoring their effect is critical for quality control and R&D alike. While the human eye is naturally fine-tuned at “change detection”^18^, machine vision is needed to meet the requirements of automated, quantitative, reliable, and high-throughput low-cost inspection. For achieving the highest possible reliability in identification and analysis of chemical vapour deposited (CVD) films and exfoliated two-dimensional materials, it is necessary that both false-positives and false-negatives are avoided.

Here we show that accurate estimates for the pixel contrasts of 2D materials can be modelled in the well-known framework of multiple Fresnel reflections, and then used as a starting point for consistent identification of 2D crystals. To reduce false-positives and false-negatives as far as possible the individual RGB (Red, Green, Blue) spectral profiles of the light source, the 2D material-SiO_2_ interface, and finally the charge-coupled device (CCD) camera are taken into account. We then present a robust methodology for reliably discriminating between 2D material flakes or films and other confounding image elements with similar contrast, such as resist residues and other contaminants. Coverage is a *de-facto* standard for characterising the quality of graphene growth and transfer, and the approach presented here provides a fast, reliable, and quantitative means of obtaining the actual coverage of transferred graphene. Finally, we demonstrate how arbitrarily large datasets can be processed for easy inspection and analysis, which greatly improves the practicality of our approach, thereby providing a fast, reliable, and highly accurate means of obtaining the numerical coverage, a quantitative indicator of the quality (e.g. coverage percentage, layer number, contamination density) of transferred CVD-based 2D material films, for arbitrarily large samples.

## Materials and Methods

### Growth and Exfoliation

CVD graphene was grown on electropolished copper foils using a commercially available Black Magic Pro CVD system. The growth recipe is based on a published recipe^19^ with an annealing phase in H_2_/Ar where CH_4_ was added as a precursor for the growth phase at 1000 °C followed by a cool down in Ar. Graphene was transferred from the copper foil to 90 nm SiO_2_ using either a standard wet etching process^20,21^ or the electrochemical oxidative decoupling transfer method^3^. Exfoliated graphene and WSe_2_ samples were made by the standard “Scotch tape” technique^22^, using low-contamination SWT 10+ tape from Nitto Denko.

### Optical Characterization

Optical microscopy was conducted with a Nikon Eclipse L200N, equipped with a programmable Prior Scientific XYZ stage and a Nikon DS-Fi2 camera (Sony ICX282AQ CCD).

Raman spectroscopy was performed with a Thermo Fisher DXRxi spectrometer equipped with a 532 nm excitation laser source using 1 mW laser power and 100x objective. We use the intensity ratio of the graphene 2D and G Raman peaks determined from fits to single Lorentzian functions^23^ to distinguish between single and bilayer graphene on SiO_2_. The *I*(2D)/*I*(G) peak intensity ratio is set to 0 in pixels where there is no detectable Raman G peak.

## Results and Discussion

### Calculating Pixel Contrast

**Figure 1:**
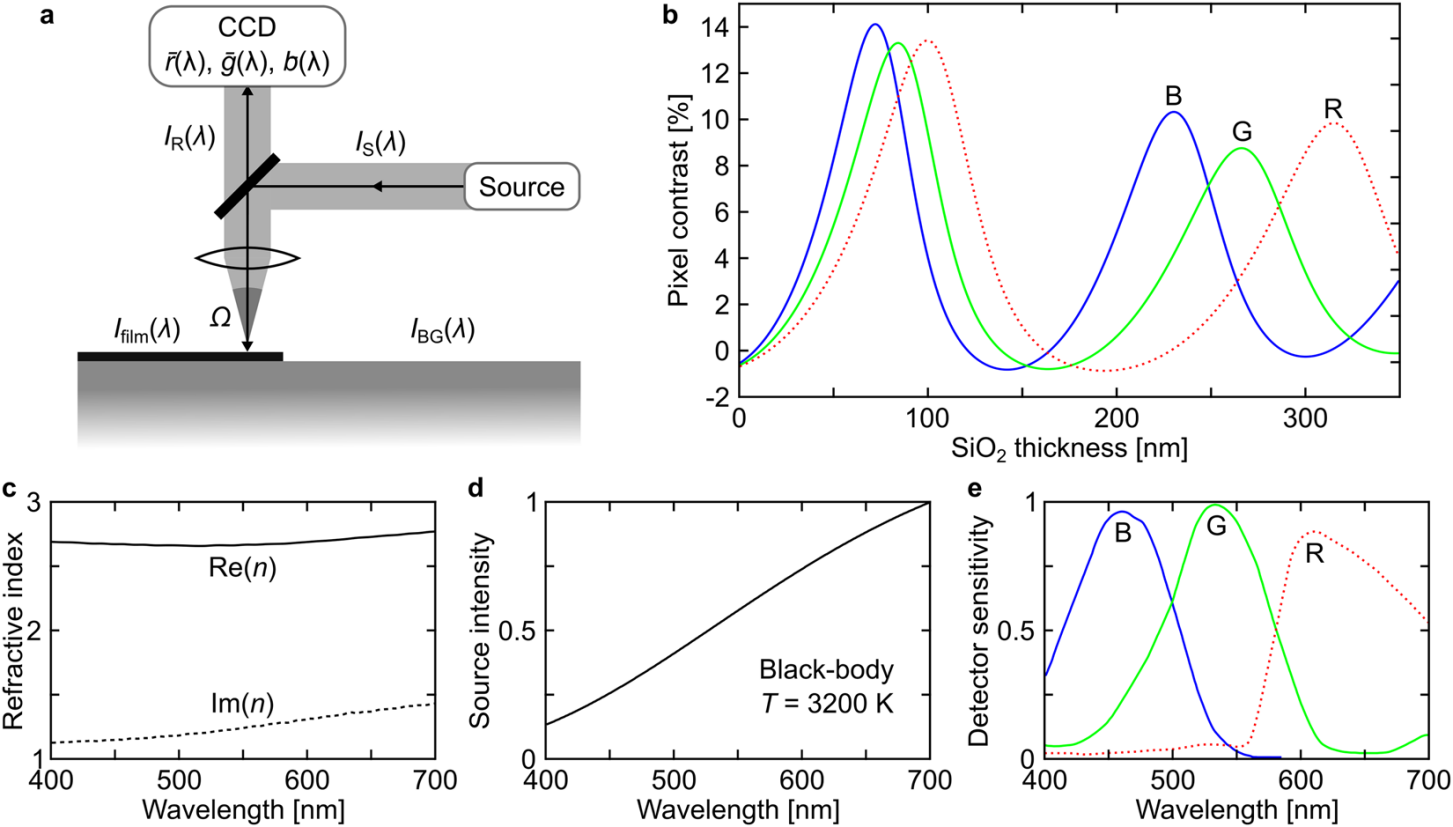
(**a**) Illustration of a typical optical setup where light from a source with spectral intensity *I*_S_(λ) interacts with the substrate and optical components and arrives at a detector as *I*_R_(λ). *I*_film_(λ) and *I*_BG_(λ) are the reflected spectral intensities from the material to be identified and the background, respectively. (**b**) Calculated red, green and blue pixel contrast of single-layer graphene on a SiO_2_/Si substrate as a function of varying thickness of SiO_2_. (**c**,**d**,**e**) Wavelength-dependent input to the contrast calculations. (**c**) Real and imaginary part of the refractive index of graphene. (**d**) Typical intensity characteristics of an incandescent light-source. (**e**) Spectral sensitivity of a Sony ICX282AQ CCD camera.

Detection of 2D materials in white-light microscopy, whether by eye or machine vision techniques, relies on a detectable difference in the intensity of reflected light from the target material and its substrate, which depends on the path of light in the optical setup. A generic schematic of machine vision or optical inspection of 2D materials in the visible regime is shown in Fig. 1(a). Here the sample, a substrate covered with 2D material regions of different lateral sizes and thicknesses in addition to a range of other particles and contamination is illuminated by a light-source with spectral intensity *I*_S_(*λ*). The reflected light from the sample, either *I*_BG_(*λ*) from the reference background or *I*_film_(*λ*) from the 2D material, is collected and focused by lenses onto a detector, which finally converts the light to red, green, and blue digital pixel intensities according to its spectral sensitivity functions $\bar{r}(\lambda )$, $\bar{g}(\lambda )$, and $\bar{b}(\lambda )$, respectively. 2D materials are commonly identified from their wavelength-dependant optical contrast, *C*(*λ*) = (*I*_BG_(*λ*) − *I*_film_(*λ*))/*I*_BG_(*λ*). In order to identify the presence of a given 2D material the corresponding pixel values of digitally captured images must be identified during inspection. To this end, the spectral intensities are substituted with the calculated or digitally captured pixel intensities. With a given light-source, reflection spectrum, and camera spectral sensitivity function, the absolute intensities of RGB pixels are given by

1a $$R={\int }_{0}^{\infty }{I}_{R}(\lambda ){I}_{S}(\lambda )\bar{r}(\lambda )d\lambda ,$$

1b $$G={\int }_{0}^{\infty }{I}_{R}(\lambda ){I}_{S}(\lambda )\bar{g}(\lambda )d\lambda ,$$

1c $$B={\int }_{0}^{\infty }{I}_{R}(\lambda ){I}_{S}(\lambda )\bar{b}(\lambda )d\lambda ,$$

where *I*_R_(*λ*) is the spectral intensity of light reaching the detector, either from the background, *I*_BG_(*λ*), or from the 2D material, *I*_film_(*λ*). The reflection spectra are functions of the thickness and refractive indices of the given material stack, along with the angular distribution of the impinging light^24^. The details of calculating the reflection spectra can be involved^25–28^, and should for critical applications include the effect of lenses and other optical elements in the specific setup. In this work, we restrict ourselves to thin-film calculations for normal incident light similar to previous work by Blake *et al*.^1^. Contrast calculations for non-normal incident light, in particular describing discrepancies between observed and expected contrast due to high numerical aperture objectives can be seen in the Supplementary Information.

With a given material and its refractive index, along with illumination and camera characteristics, the pixel intensities for, *e.g*. graphene and its substrate can now be calculated and used to obtain a set of (pixel) contrast curves, as shown in Fig. 1(b). Here, we have used the ellipsometrically measured refractive index of single-layer graphene, Si, and SiO_2_^29–31^, the intensity profile of an incandescent light source (3200 K black-body spectrum) and a typical CCD response function (see methods) as shown in Fig. 1(c–e), which yields quantitative values of the contrast of 2D materials in digital optical images. As is well known^1^, the optimal thickness of SiO_2_ for simultaneous high contrast across the visible spectrum is 90 nm. Similarly, we find the highest RGB pixel contrasts for 90 nm SiO_2_, where the green contrast is slightly higher than the red and blue contrasts, which is opportune for manual identification, due to the high sensitivity of the human eye to green light^32^. These contrast values then serve as the starting point for a machine vision approach to identify the presence of 2D materials in digital images. Any color shift introduced by the optical path of the imaging system will affect all images uniformly, and will not affect contrasts so as to cause misidentification of layer number (in effect a contrast inversion) in any well designed system. Through manual calibration such as white balancing small color shifts can also be well accounted for.

**Figure 2:**
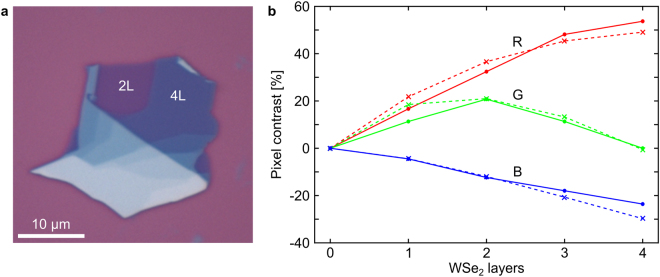
(**a**) Optical image of exfoliated WSe_2_ crystal with different number of layers on 300 nm SiO_2_. (**b**) Contrast of WSe_2_ as a function of number of layers on 300 nm SiO_2_. Crosses are calculated contrasts based on the wavelength dependent refractive index of WSe_2_. Dots are extracted from digital optical images with red, green and blue channels giving three separate values of contrasts for each thickness.

There is a lack of data and consensus on the layer-number dependent values of the optical constants of other 2D materials, such as most of the transition metal dicalchogenides, in particular where changes in these constants associated with the transition from indirect to direct bandgap structures are expected for the monolayer. We note however that for published systems such as WSe_2_ the assumption that the optical constants do not change from bulk to monolayer is not coarse enough to cause a misidentification of the monolayer as a bilayer, and can therefore be applied in these cases. Figure 2 shows the pixel contrasts extracted from optical images of exfoliated WSe_2_ compared to values calculated from the wavelength dependent refractive index of bulk WSe_2_^33^, and a comparison between pixel constrasts obtained with monolayer and bulk refractive indicies can be found in the Supplementary Information.

The experimentally and theoretically determined contrasts are in good agreement despite changes in the electronic band structure when going from single to few-layer WSe_2_^34^. This shows that the expected RGB contrast can be successfully calculated for a wider range of 2D materials with varying thickness than simply graphene (Fig. 1) and hexagonal boron nitride (hBN)^2,4,35,36^. We caution that the assumption of bulk optical constants for monolayers of a given material is not likely to be universally accurate, and must be tested for validity for new materials accordingly.

### Quantitative Identification

**Figure 3:**
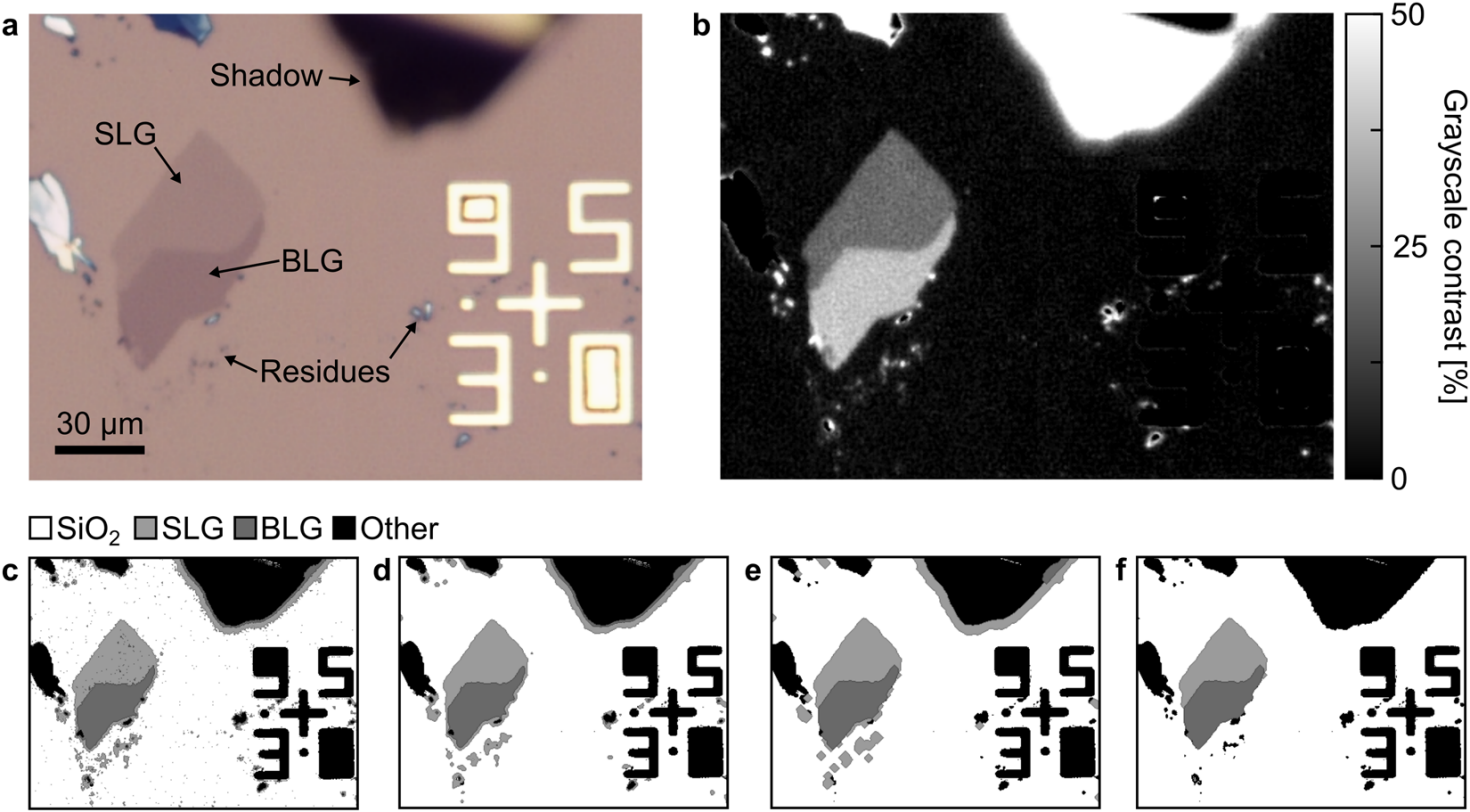
(**a**) Optical image of exfoliated graphene on 300 nm SiO_2_. The sample contains typical features of an optical image of an exfoliated sample, such as single- and bi-layer graphene, graphite, and residues, a shadow from bulk graphite, and optical alignment marks. (**b**) Map of the positive grey-scale contrasts in the image. (**c**) Segmented image where every pixel has been categorized either as SLG, BLG, SiO_2_, or other, using only the expected contrast values. (**d**) Segmented image after edge-preserving median filtering, and (**e**) after erode and dilate filtering. (**f**) Segmented image after contour filtering, resulting in a >99% pixel-for-pixel correspondence between expected and automatically detected pixels.

Once the expected contrast of a 2D material on a given substrate is known, it is in principle possible to select the corresponding pixels of a digital image, thus identifying regions where any 2D materials are located. Figure 3(a) shows a typical optical image of exfoliated graphene on SiO_2_ which we use to illustrate the effect of the individual steps of our method. The image contains several different visible elements: a region of single-layer graphene (SLG), bi-layer graphene (BLG), alignment marks in gold, residues (tape adhesive and graphitic fragments), and a shadow from a nearby folded graphite flake. While the presence of SLG is immediately visible by optical inspection, many other regions of the area show contrast close to the expected contrast of single-layer graphene. This is evident from the corresponding map of the grey-scale contrast, Fig. 3(b), where the graphite shadow and residues have similar grey-scale contrast as the single-layer graphene, relative to the substrate, and therefore could be easily confused if a grey-scale analysis was used.

Grey-scale images, sometimes converted from RGB images^37^, can be used to identify 2D materials^26,38,39^. However, since RGB images retain a degree of spectral information, significantly better results can be achieved by individually treating each RGB channel^40^. This is especially evident for materials such as hBN where the contrast changes sign over the visible optical range^2^, and therefore the grey-scale contrast can be zero even when the individual RGB contrasts are not.

By filtering the image based on a certain range of RGB contrasts around the value expected for the different 2D materials and layer thicknesses to be identified, we arrive at an image with clear regions of SLG, BLG and SiO_2_, as seen in Fig. 3(c), where any remaining pixels are designated as “other”. While outlining most of the SLG and BLG, the image still reveals numerous missing pixels in the interior of the graphene flakes and false-positives from the surrounding residues and graphite shadow^40–42^.

First, we apply a median filter to the image, as seen in Fig. 3(d). This effectively removes impulse noise while keeping edges sharp^43,44^. With the impulse noise removed, the full interior of the SLG and BLG can be identified.

Next, we apply consecutive erode and dilate filters, as seen in Fig. 3(e). Erode and dilate, from mathematical morphology, effectively removes or adds pixels around a shape in binary images^45^. By applying these filters consecutively, small and thin features, such as small particles, tape residues, and other contamination, are completely removed by the first erode filter, while larger structures are mostly preserved by the subsequent dilate filter. This effectively removes the smallest of the false-positive features but does slightly soften sharp features such as corners. It should be noted, however, the dilate and erode filters can have the effect of smoothing out sharp features such as corners, as seen in Fig. 3(e), and is further discussed in the Supplementary Information. This slight softening is preferable to returning a large number of few-pixel sized false positives since the structure of sharp corners can be effectively reconstructed by polygonal contour fitting of the shape boundary in later steps, the processing overhead relating to large numbers of false positives is eliminated and that very small areas of material are typically of limited technological interest or not the material of interest.

Finally, we add an edge-detection algorithm to mark up all identified regions by their polygon-approximated contour outlines^46^. Each identified contour is now represented by *n*-sided polygons, described by a list of *n* pixels around the perimeter. From these contours, a range of shape-dependent quantities can be directly extracted, such as area and perimeter; and quantities such as circularity, pseudo-width, and pseudo-length can be derived from these. These quantities can now be used as a final filtering criterion, such as filtering out remaining small-area particles from their contour-area, or eliminating shadows from graphite pieces due to their crescent-shape leading to a low contour-circularity. As for the edge-detection, the previous removal of noise and false-positives strongly decreases the computation time for the contour-filtering, which is only needed on a small number of items. This procedure results in a highly reliable identification, as seen in Fig. 3(f), with a low computational cost. Furthermore, all of the steps described can be implemented with well-documented algorithms from highly optimised image processing libraries, making identification of 2D materials straightforward to implement, high-throughput, and scalable.

### Large-scale coverage analysis

To demonstrate large-scale coverage analysis, which is an important aspect of 2D materials quality-control, a transferred CVD graphene sample was imaged with the quantitative optical method described above and compared with spatial maps of relevant micro-Raman spectroscopic parameters. The sample was grown on Cu foil and transferred to SiO_2_ by wet etching (see Methods) and exhibits a certain fraction of macroscopic defects, bi-layer areas (either folded or secondary growth), and contamination predominantly from polymer residues.

**Figure 4:**
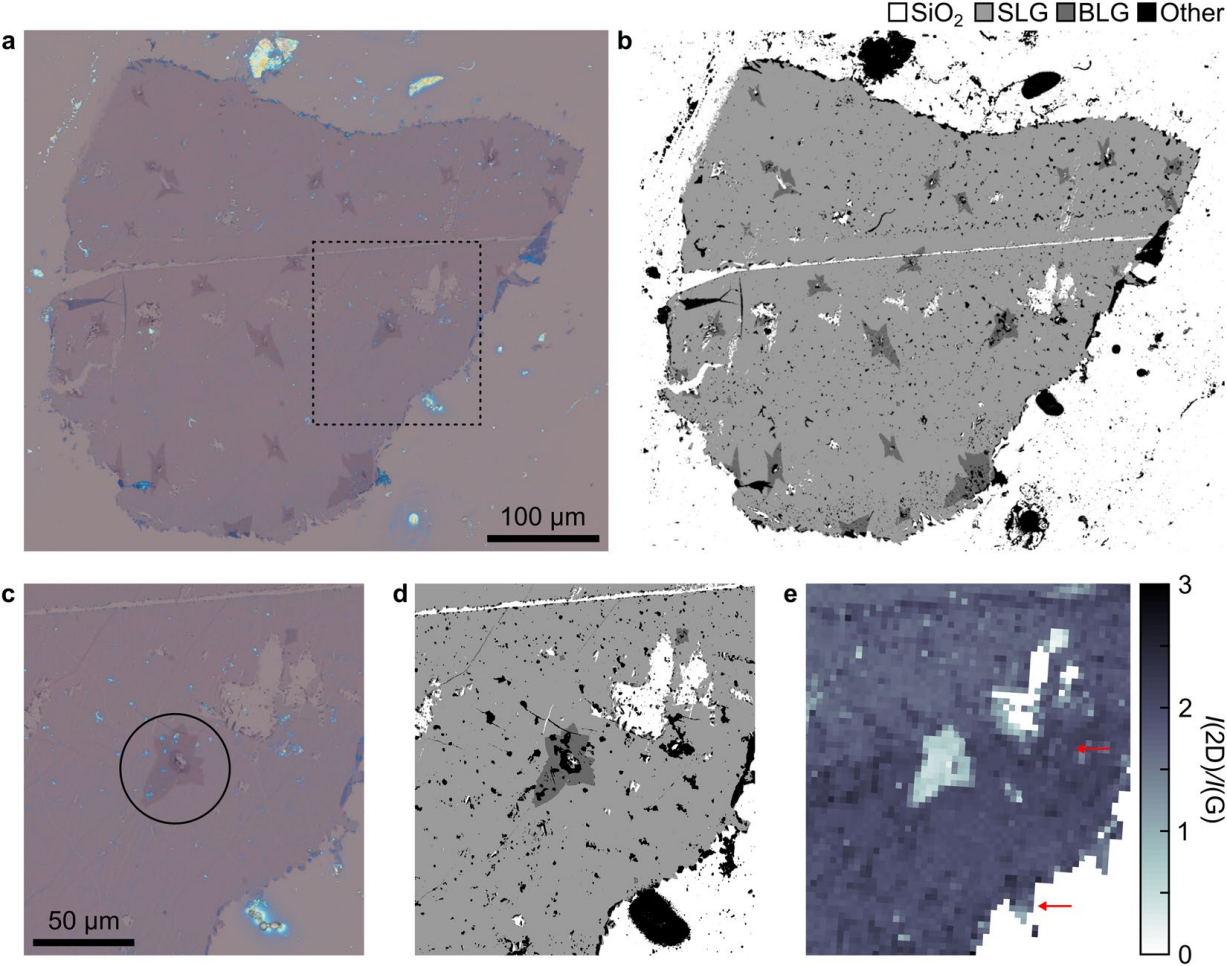
Comparison between optical, coverage and Raman maps of CVD graphene transferred by wet etching to 90 nm SiO_2_. (**a**) Optical map of graphene consisting of several images stitched together. (**b**) Coverage map of the sample in (a) with colour codes for SiO_2_, SLG, BLG, and other. (**c**) Zoom-in on the area inside the dotted square in (a) with the corresponding coverage map in (**d**) showing the detail level of the optical and coverage maps. The circle in (a) highlights an area with BLG surrounding a small region with three layer graphene and SiO_2_. (**e**) Raman map of the *I*(2D)/*I*(G) peak intensity ratio for the area shown in (c) and (d).

Large-area optical maps of the graphene were generated by stitching together the acquired images, Fig. 4(a). Quantitative coverage analysis was used to identify regions of SLG and BLG, which are shown in Fig. 4(b) as light grey and dark grey pixels, respectively. Pixels with zero contrast can be ascribed to SiO_2_, and are shown as white pixels. Finally, pixels categorised as “other” fall outside any of the other categories, and represent areas with three graphene layers or more, as well as areas contaminated by polymer residues or other remnants from the growth and transfer processes. The resulting coverage map is shown in Fig. 4(b) and highlights contamination and defects in the transfer which might otherwise not be clearly visible. The coverage can subsequently be calculated as the fraction of pixels identified as graphene and the total number of pixels in a defined region of the sample. For the sample shown in Fig. 4(b) the coverage of SLG is 48.6%, BLG 2.6%, and “other” is 6.8%.

By examining a specific area more closely, we can highlight the detailed level of the coverage mapping in Fig. 4(c,d). Small holes and lines of missing graphene from the growth and/or transfer process are precisely determined, within the limit of the optical system used. A BLG region around a small three-layer graphene area in the centre of the image (black circle) is also well represented in the coverage map as bilayer graphene and “other”, respectively. The bright blue dots on the sample are polymer residues remaining from the transfer method, also represented as “other” in the coverage map. Based on an optical image, it cannot be excluded that there is graphene beneath areas covered by polymer residue. Therefore, the numerical graphene coverage determined with this method strictly serves as the lower bound for the coverage, or specifically the coverage of SLG without macroscopic contamination. Figure 4(d) clearly shows the regions with SiO_2_, SLG, and BLG, as well as regions with everything else marked as black. Figure 4(e) shows the Raman spectroscopic *I*(2D)/*I*(G) peak intensity ratio, for comparison with Fig. 4(c,d). It is clearly seen that *I*(2D)/*I*(G) is ~2 in the areas discerned as SLG in the coverage map, while *I*(2D)/*I*(G) drops to ~1 for the BLG region inside the black circle, as expected^47^. As the red arrows in Fig. 4(e) indicate, the Raman analysis shows graphene in areas that the quantitative optical method categorises as “other”, which confirms the presence of graphene under macroscopic contaminants. The Raman mapping confirms that the coverage analysis gives a lower bound of the 2D material coverage in cases where the sample is contaminated.

The technique presented here is thus a way to systematically determine not only the presence and number of graphene layers, but also whether it is free from or covered by residues or other contamination, or contains macroscopic defects such as folding and tearing.

### Scalable inspection using data-pyramids

Since handling of numerical data and images in the giga-pixel range and beyond is outside the capabilities of most commercially available software and analysis systems, we use here an approach that draws inspiration from online services for presenting geo-spatial data that allows visualization and numerical analysis to be carried out on arbitrarily large data sets with very moderate computing power.

**Figure 5:**
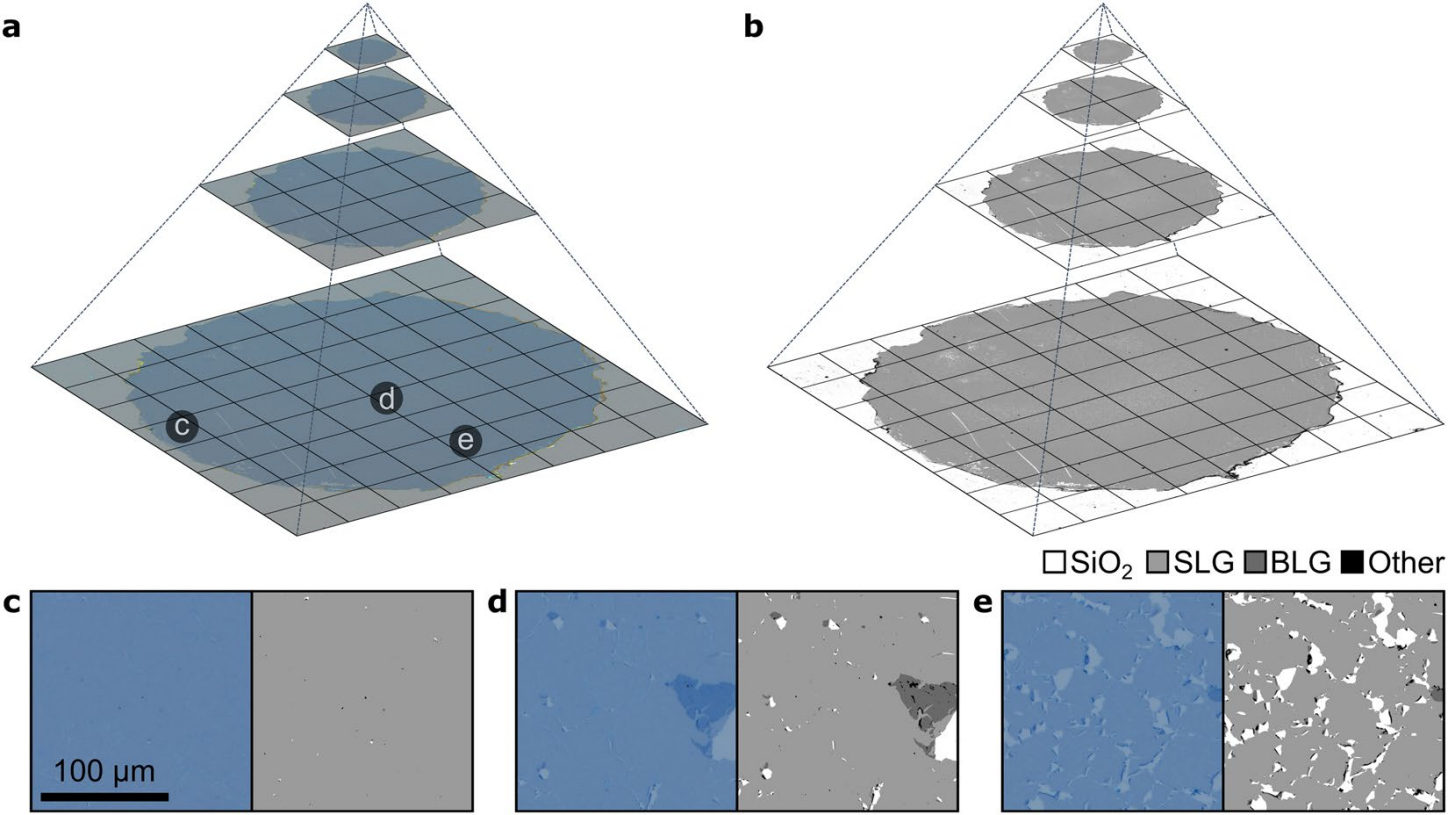
(**a**) Representation of the data-pyramid of a 12 mm wide circular sample of CVD graphene transferred to 90 nm SiO_2_ using oxidative decoupling transfer, while (**b**) is the corresponding data pyramid from coverage analysis. The contrast of images is slightly enhanced to increase visibility. In the left and right sides of figure (**c**–**e**) is shown the raw images and digitally detected counterparts, respectively, of selected areas in the data-pyramid highlighted in (a). The dataset is 16·10^9^ (16 giga) RGB pixels.

Data pyramids are hierarchical sets of data obtained by progressively subsampling which in the present case have spatial extent. Data pyramids only show and treat data at a resolution that is relevant to the user and the spatial scale at which the data is viewed, rather than the full resolution. This data-structure allows correlation of several data sets with shared coordinates, highly efficient numerical analysis, and handling of essentially arbitrarily large data sets. The bottom level of the pyramid contains full-resolution data while each subsequent level is sampled to half the side-length (and thus a quarter of the resolution), as illustrated in Fig. 5(a,b). Data pyramids are the underlying principle of the smooth, streaming experience in online map services, which supports seamless zooming spanning more than 10 orders of magnitude in scale^48^.

Every move up in the pyramid results in the side-lengths being halved. The total number of levels, *l*_max_, is thus given by how many times we can down-sample the longest side of the full-resolution level, given by,

2 $${l}_{max}=\lceil {\mathrm{log}}_{2}({\rm{\max }}({x}_{max},{y}_{max}))\rceil .$$

The total size of the dataset thus increases as levels are added on top of the bottom (full resolution). As each subsequent level in the data-pyramid is one quarter the size of the previous, the maximum possible increase in size of the dataset is given by

3 $$\frac{1}{4}+\frac{1}{16}+\frac{1}{64}+\cdots ={\sum }_{n=1}^{\infty }\frac{1}{{4}^{n}}=\frac{1}{3}.$$

Building a data-pyramid thus increases the total size of the dataset by no more than a factor of 4/3, a modest amount considering that it allows for easy inspection and numerical processing of arbitrarily large datasets. After the data-pyramid has been generated, virtually no processing power is needed to inspect the data, as the (next) required level is simply (pre)loaded into memory.

Given that only a subset of the full data is ever accessed, it is possible to do on-demand real-time processing of the data. Most types of trivial data analysis or image treatment of the accessed data, such as contrast enhancement, statistical analysis, etc., are computationally inexpensive to perform. We present an example of a zoomable map of graphene including on-demand processing options using correlated data pyramids, which can be found online^49^.

The data pyramids are generated from a large number of images collected with an optical microscope with a standard motorised stage. An overlap of ~5% for adjacent images is in most cases sufficient to generate near-seamless, continuous images of arbitrarily high resolution. Each data pyramid is generated by successively down-sampling the images, starting with the full-resolution data, and repeating this process until the final level is 1 × 1 pixel. This is illustrated in Fig. 5, which shows four successive levels of pyramids for optical and coverage data (Fig. 5(a,b)), as well as images for three representative areas in the highest resolution layer (Fig. 5(c–e)). In principle, the only limit to the size of the data pyramids is data collection and storage, which even with modest desktop solutions can reach tera-pixel resolution and beyond.

## Conclusion

In conclusion, we have presented an accurate, robust and fully automatic method for identification of graphene or other 2D materials, which successfully rejects false-positives and false-negatives. The accuracy of the quantitative coverage measurements of graphene has been shown to depend on the degree of contamination, but the method give a reliable lower-bound estimates of the coverage.

The method employs a combination of the RGB spectral fingerprints from all relevant parts of the light path; the light source, the detector, the two-dimensional film and the substrate, and refines the coverage using a combination of well-established image processing methods. Data pyramids are computationally and resource efficient for an arbitrarily large dataset of images, and serve as an optimal platform for quantitative and qualitative analysis of 2D material films on arbitrarily large areas, with a resolution ultimately limited by the microscope optics used. The method can be used for automated identification and analysis of exfoliated materials, which is a tedious and error-prone procedure when performed manually, for in-line quality control of CVD graphene, or as an effective means of monitoring improvements in 2D material transfer and growth methods. Such data sets form a basis for data mining and deep statistical analysis of results generated across long time spans and between disparate characterisation techniques and provide the possibility to extract trends and correlations that would not be apparent from manual inspection of isolated regions alone.

## Electronic supplementary material

Supplementary information
