# Supplementary material for: Quantitative optical mapping of two-dimensional materials
Source: Sci Rep. 2018 Apr 23;8:6381. doi: 10.1038/s41598-018-23922-1 (PMC5913130; doi:10.1038/s41598-018-23922-1)
Supplement: Supplementary file 1 — Supplementary information [file 41598_2018_23922_MOESM1_ESM.docx]

**Supplementary information: Quantitative optical mapping of two-dimensional materials**

Bjarke S. Jessen^1,2,*^, Patrick R. Whelan^2,*^, David M. A. Mackenzie^1,2^, Birong Luo^2^, Joachim D. Thomsen^2^, Lene Gammelgaard^2^, Timothy J. Booth^1,2^ & Peter Bøggild^1,2, †^

^1)^ *Center for Nanostructured Graphene (CNG), Technical University of Denmark, DK-2800, Kgs. Lyngby,* *Denmark*

^2)^ *Department of Micro- and Nanotechnology (DTU Nanotech), Technical University of Denmark, DK-2800, Kgs. Lyngby,* *Denmark*

^*)^ These authors contributed equally to the work

^†)^ Corresponding authors: Bjarke S. Jessen, [bjarke.jessen@nanotech.dtu.dk](mailto:bjarke.jessen@nanotech.dtu.dk), and Peter Bøggild, [peter.boggild@nanotech.dtu.dk](mailto:peter.boggild@nanotech.dtu.dk)

# **Pixel contrasts when including numerical aperture**

In the model of the main text for calculating the pixel contrasts, we consider incident light, i.e. light arriving perpendicular to the interface. However, in most practical applications light will impinge on the surface with a certain angular distribution, usually Gaussian. In free-space applications, this angle, $\theta$, is given by the numerical aperture (NA) through the relation

$\mathrm{NA}=\sin(\theta)$.

Here we show the effect in the red, green, and blue (RGB) pixel contrasts when including NA. The spectral intensity of light reaching the detector, $I\left( \lambda\right)$, is the product of the source spectral power distribution, $I_{S}\left( \lambda\right)$, and the solid-angle integrated reflection spectra, $I_{R}\left( \lambda,\Omega\right),$ according to

$I\left( \lambda\right)=I_{S}\left( \lambda\right)\iint_{S} I_{R}\left( \lambda,\Omega\right)W\left( \Omega\right)d\Omega$,

where $W\left( \Omega\right)$ is a weight function describing the solid-angle distribution of light. Assuming the incident light distribution to be azimuthally uniform, the weight function can be estimated as a Gaussian with expected value $\mu=0,$ and standard deviation $\sigma=\theta$^1^.

Figure S1 shows the resulting RGB pixel contrasts of single-layer graphene as a function of NA and SiO_2_ thickness. The inclusion of NA can be seen to have little impact on the contrast values below NA = 0.4, corresponding to typical magnifications in the range of 20x. This is further evident in Figure S2, where we show the pixel contrasts as a function of NA for the typical SiO_2_ thickness of 90 nm. At NA values below 0.4, the relative error (i.e. the error from not including NA in the calculations), stay below 10%, but increases at higher NA values, highlighting the importance of considering NA in high-magnification applications.

Figure S1: Calculated red, green, and blue pixel contrasts of single-layer graphene as a function of numerical aperture (NA) and thickness of SiO_2_. At higher NA values, typically meaning higher magnification, the change in contrast becomes higher for a given SiO_2_ thickness.

Figure S2: (left) Calculated pixel contrast of single-layer graphene on 90 nm of SiO_2_, as a function of numerical aperture (NA). At higher NA values, the pixel contrasts starts to deviate from the case of incident illumination. (right) The relative error in calculated contrast values of NA is not considered. At NA values below 0.4, typically corresponding to 20x magnification, the relative error stays below 10%. At higher magnifications, the relative error becomes significantly larger, making the inclusion of NA necessary for accurate calculations.

# **Pixel contrast of WSe_2_ for mono-layer vs bulk optical constants**

Unlike graphene, the optical constants of most other 2D materials, such as WSe_2_, change significantly depending on the number of layers. Here we calculate the pixel contrasts of single-layer WSe_2_, using both the reported bulk and mono-layer optical constants. We use the measured dielectric functions from^2^, to calculate the complex refractive index,

$N=n+i\kappa$ ,

where $n$ is the refractive index, and $\kappa$ is the extinction coefficient, according to

with and, and being the real and imaginary part of the complex dielectric function, respectively.

In Figure S3 we show the calculated pixel contrast for a single-layer (0.67 nm thickness) of WSe_2_, as a function of SiO_2_ thickness, using both the bulk and single-layer optical constants. Both peak-positions and absolute contrasts are seen to be in close agreement. Since the difference between the two approaches gives errors significantly smaller than the contrast difference change when moving from N to N+1 layers, we argue that even using bulk optical constants can serve as an excellent starting point for estimating the expected pixel contrasts of a given 2D material, and the subsequent quantitative identification scheme given in the main text.

Figure S3: Calculated pixel contrast of mono-layer WSe_2_ as a function of SiO_2_ thickness, for normal incident light. Solid lines represent calculations using mono-layer optical constants, while dashed lines represent calculations using the bulk optical constants.

# **Comparative analysis of optical images to coverage maps**

The optical coverage mapping determines the pixel-by-pixel layer coverage including the number of layers of graphene based on a spectrally resolved contrast analysis, where the spectral fingerprints of both light source and image sensor are taken into account, as well as image processing algorithms that reduce noise and artefacts.

The identification of coverage and number of layers depends on a number of factors, including the precise settings for the filters, the precision and time-stability of the spectral characteristics of the image sensor as well as the light source, the finite diffraction limited resolution of high magnification optical microscopes, possible color and image distortions by the optics and many more. In practice the image processing filters should be calibrated to give the best possible agreement between the manual, optical identification (or alternatively by high resolution Raman spectroscopy) with the numerical analysis following coverage mapping.

Thus, the most meaningful way to discuss the quantitative uncertainty of the method is by comparing the number of incorrectly categorized pixels, either as false negatives (no graphene, where there is), false positives (graphene, where the is no graphene), or incorrect discrimination of 1 and 2 (or more) layers, with the total number of pixels.

This number is highly dependent on the type of sample, the density and distribution of graphene, and the amount of contamination. We show in Figure S4 images of chemical vapor deposited graphene after incomplete transfer to a silicon dioxide substrate, with a large number of gaps, tears, holes and folded graphene (where the single layer has folded up on itself, which in terms of contrast is identical to a bilayer). In addition, polymer residues from the transfer are scattered on the graphene surface. This represents a worst case scenario, and therefore should give a lower bound for the uncertainty. On the other hand, these samples have just single layers (self-terminating CVD growth on copper) and double layers (from folding) and are therefore straightforward to interpret, and therefore useful for benchmarking the figure.

Panels (a) and (d) show 80x80 µm 1560x1560 pixel optical microscope images of two CVD graphene samples. Panels (b) and (e) show the corresponding coverage maps, after quantitative analysis. White color corresponds to “no graphene”, light grey to “single layer”, dark gray to “double layer” and black to anything else, i.e. multilayers (more than 2) and contamination.

Panels (c) and (f) show the subtracted images. The dark lines represent the fringes of the gaps, where the coverage analysis and the contrast as determined from the optical image do not agree (difference in contrast larger than 20%). The dark pixels represent less than 3% of the area for both panel (c) and panel (f). These numbers are typical for dirty and damaged samples, and can be both larger or smaller depending on the distribution of the crystals on the sample.

This analysis shows that while a single number for the uncertainty is not possible to give, the method is able to correctly associate the pixel contrasts to areas covered by single layer graphene, double layer graphene and other, even in highly damaged regions, to within a few percent.


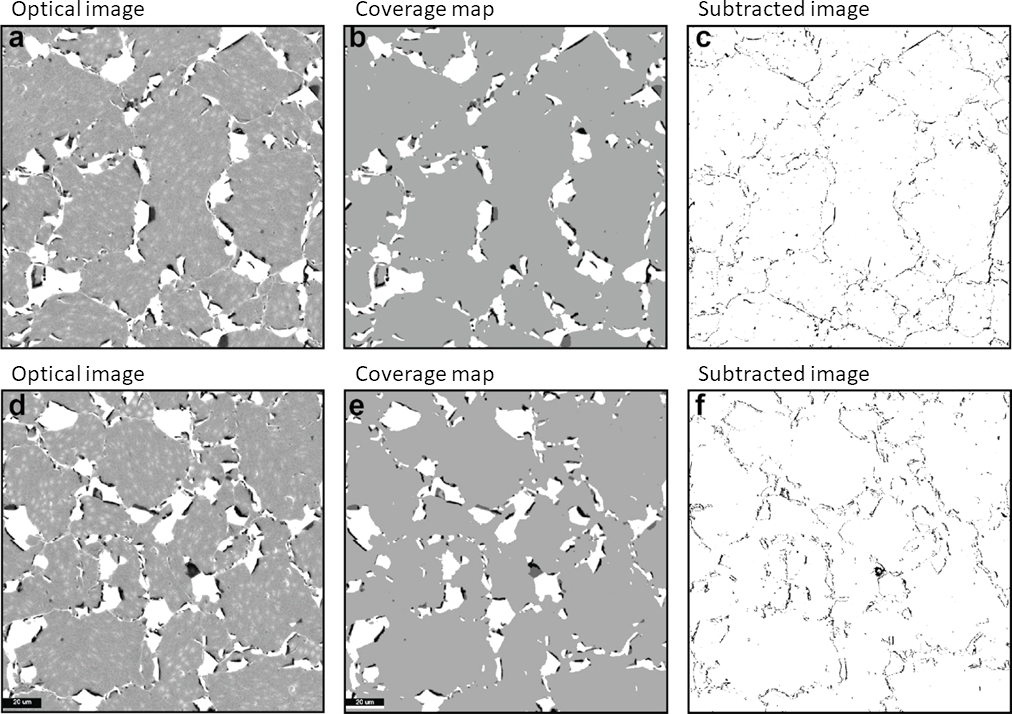


Figure S4: (a,d) Optical images of CVD graphene transferred to 90 nm SiO_2_ with corresponding coverage maps (b,e). The differences between the optical images and coverage maps are shown in (c,f).

**REFERENCES**

1. Bruna, M. & Borini, S. Assessment of graphene quality by quantitative optical contrast analysis. *J. Phys. D. Appl. Phys.* **42,** 175307 (2009).

2. Li, Y. *et al.* Measurement of the optical dielectric function of monolayer transition-metal dichalcogenides: MoS_2_, MoSe_2_, WS_2_, and WSe_2_. *Phys. Rev. B* **90,** 205422 (2014).
